# An International Survey of Aquaponics Practitioners

**DOI:** 10.1371/journal.pone.0102662

**Published:** 2014-07-16

**Authors:** David C. Love, Jillian P. Fry, Laura Genello, Elizabeth S. Hill, J. Adam Frederick, Ximin Li, Ken Semmens

**Affiliations:** 1 Johns Hopkins Center for a Livable Future, Johns Hopkins University, Baltimore, Maryland, United States of America; 2 Department of Environmental Health Sciences, Bloomberg School of Public Health, Johns Hopkins University, Baltimore, Maryland, United States of America; 3 University of Maryland Extension, Baltimore, Maryland, United States of America; 4 Maryland Sea Grant College, University of Maryland College Park, Maryland, United States of America; 5 Department of Biostatistics, Bloomberg School of Public Health, Johns Hopkins University, Baltimore, Maryland, United States of America; 6 Agriculture and Natural Resources Unit, West Virginia University Extension Service, Morgantown, West Virginia, United States of America; The Ohio State University, United States of America

## Abstract

Aquaponics, a combination of fish farming and soilless plant farming, is growing in popularity and gaining attention as an important and potentially more sustainable method of food production. The aim of this study was to document and analyze the production methods, experiences, motivations, and demographics of aquaponics practitioners in the United States (US) and internationally. The survey was distributed online using a chain sampling method that relied on referrals from initial respondents, with 809 respondents meeting the inclusion criteria. The majority of respondents were from the US (80%), male (78%), and had at least a high school degree (91%). The mean age of respondents was 47±13 years old. Most respondents (52%) had three years or less of aquaponics experience. Respondents typically raised tilapia or ornamental fish and a variety of leafy green vegetables, herbs, and fruiting crops. Respondents were most often motivated to become involved in aquaponics to grow their own food, for environmental sustainability reasons, and for personal health reasons. Many respondents employed more than one method to raise crops, and used alternative or environmentally sustainable sources of energy, water, and fish feed. In general, our findings suggest that aquaponics is a dynamic and rapidly growing field with participants who are actively experimenting with and adopting new technologies. Additional research and outreach is needed to evaluate and communicate best practices within the field. This survey is the first large-scale effort to track aquaponics in the US and provides information that can better inform policy, research, and education efforts regarding aquaponics as it matures and possibly evolves into a mainstream form of agriculture.

## Introduction

Aquaponics is the mutually beneficial integration of hydroponics (*e.g.,* soilless systems for crop production) and aquaculture (*e.g.,* aquatic animal farming) to simultaneously produce plant and animal products. In an aquaponic system, aquatic animals excrete waste, bacteria convert the waste into nutrients, and plants remove the nutrients and improve water quality for the aquatic animals. A brief history of hydroponics and aquaculture helps provide a context for how and when aquaponics was established as a field.

Aquaponics applies methods developed by the hydroponics industry. The development of hydroponics can be traced to work by Dr. William Gericke at the University of California in 1929 [1]. Chemical salts dissolved in water are the source of nutrients in hydroponics systems. Most hydroponics operations are performed in controlled environment facilities, such as greenhouses, which were developed following World War II as an industrial approach to intensively grow food crops [2]. The introduction of plastics in the 1940s, and particularly clear polyethylene as a cover for greenhouses, was an important development. It is common for commercial aquaponic operations to use greenhouses and controlled-environment agriculture methods to increase crop production yields [3], essentially drawing on methods developed by hydroponics practitioners [4].

Aquaponics was also influenced by work in the early 1970s by aquaculture researchers who experimented with raising fish in land-based tanks with continuously recycled water (*e.g.,* recirculating aquaculture systems or RAS). A major challenge for recirculating aquaculture was the accumulation of nitrogen compounds, a potentially toxic by-product of fish waste [5], [6]. Investigators experimented with soilless plant systems as a means of treating fish waste and removing nitrogen compounds [7]–[10], which marked the beginnings of contemporary aquaponics. Engineers have since developed a variety of biofilters to treat fish waste that do not rely on plants [11]. The fact that aquaponic systems improved water quality and produced a second profit center, in the form of edible plants, is what distinguishes aquaponics from other forms of recirculating aquaculture.

The development of aquaponics was also influenced by the sustainable agriculture movement. The concept of farming in ways that mimic natural systems, known as permaculture, has been practiced for thousands of years, but was first codified by researchers in the mid-1970s in Australia [12]. In the late 1970s and early 1980s, Ron Zweig, John Todd, John Wolfe, and others at the New Alchemy Institute applied permaculture methods to aquaculture [13] and later experimented with linking hydroponics and aquaculture [14].

Additional refinements in aquaponics where prompted by university investigators seeking to establish aquaponics as a viable agricultural enterprise. In the 1980s, Mark McMurty adopted a flood-drain method for watering crops in sand media beds [15], [16]. Later, in the 1990s and 2000s, Dr. James Rakocy and other investigators documented the commercial productivity of aquaponics, developed deep-water hydroponics, and led a popular training course at the University of the Virgin Islands [17]–[20]. As this knowledge spreads to other locations, it continues to evolve and broaden aquaponic designs and practices [21].

Aquaponics is touted as a form of sustainable agriculture because it mimics natural systems, is water efficient, and has fewer environmental impacts than some forms of aquaculture [22]. Aquaponic systems exist at a variety of scales and for different uses: personal use or as a hobby, for community and economic development [23], as a teaching tool in science education [24], or as a means of increasing food production in urban settings where opportunities for conventional agricultural production is limited due to environmental contamination and space limitations [25].

In 2010, one expert estimated that between 800 and 1,200 home aquaponic systems and 1,000 school aquaponic systems existed in the United Sates (US) [26]; however, no peer-reviewed published studies have attempted to confirm or refine this estimate. To our knowledge, aquaponics has not been part of the comprehensive census of US commercial aquaculture or agriculture performed by the US Department of Agriculture (USDA) [27]. Therefore, major gaps exist in our knowledge of who is practicing aquaponics and where these facilities exist.

This study was conducted to fill this research gap by documenting the production methods, experiences, motivations, and demographics of aquaponics practitioners in the US and internationally using an online survey. This paper describes initial findings from all survey respondents, and future manuscripts will provide greater detail regarding the specific categories of commercial, education, and hobby aquaponics practitioners.

## Methods and Materials

### Ethics statement

The study was reviewed by Johns Hopkins University School of Public Health Institutional Review Board (IRB No: 00005088), which determined it to not be human subjects research. The survey contained a cover page providing an explanation of the study and a consent question that needed to be answered before participants could begin the survey. To ensure the anonymity of the respondents, personal identifiers such as name, e-mail address, physical address, and organization name are not presented in any reports using these data.

### Survey development and implementation

After reviewing the literature, it was determined that no suitable survey tools existed to collect information on production practices and attitudes of individuals engaged in aquaponics. The authors developed a new survey instrument using previously described methods for internet surveys [28] and after reviewing similar agriculture surveys, such as the USDA Census of Aquaculture [27]. The authors drafted survey questions and pretested them for comprehension and content with 10 persons who were either experts in or practitioners of aquaponics. They were representative of groups targeted in the survey (*i.e.,* commercial farmers, educators, hobbyists, and non-profit organizations). The survey was piloted among the pretest group, and then the final survey was distributed to the study population using a web-based survey platform (Qualtrics, Provo, Utah). The survey opened on June 25, 2013 and closed on October 1, 2013. The survey codebook is presented in Appendix S1.

The survey was distributed by the study authors and by partner organizations using a chain sampling method (*i.e.*, referral or snowball sampling) to increase reach. This sampling method relied on eighteen partner organizations to distribute the survey to their members or subscribers using their own preferred means of communication. Common modes for recruitment were e-mail listservs, online newsletters, direct email, and social media posts (*i.e.*, Facebook, Twitter). These communications included a link to the survey website and author-generated text describing the study. Partner groups were asked to send a reminder message three weeks after the initial recruitment notice. Participants were encouraged to share the survey with their contacts in the aquaponics world.

One of the authors (DCL) attended two aquaponics conferences (the 2013 Aquaponics Association Conference, Tucson, AZ, USA; the International Aquaponics Conference, Stevens Point, WI, USA) before and during the study period to describe the study and collect e-mail addresses of potential survey participants. Contact information for over 365 potential survey participants was collected at these two conferences. Mail Chimp (Atlanta, GA) was used to send a recruitment email and reminder email, if applicable, to these individuals or organizations.

The survey inclusion criteria were: 18 years of age or over; can read English; completed the survey; and had operated and maintained an aquaponics system in the previous 12 months. A single response per organization was requested. The incentive for participation was a lottery drawing among survey respondents to win one of four $75 gift cards.

### Data analysis

Data from the survey software (Qualtrics, Provo, UT) were exported and analyzed in Excel (Microsoft, Redmond, WA) or SPSS (IBM, Armonk, NY), and figures were produced in Prism (v5, GraphPad, La Jolla, CA). T-tests were conducted to compare respondent demographics by sex, with significance set at an alpha of 0.05. Error was reported as standard deviation.

## Results

### Survey responses

A total of 1,293 respondents began the survey and 84% of respondents (n = 1084) completed the survey. Of these, 809 respondents met the inclusion criteria for the study. Because chain sampling was used, the response rate could not be calculated.

### Demographics

Survey respondents demographics are presented in Table 1. Over three quarters (78%) of respondents were male. The mean age of respondent was 47±13 years old, which did not differ by gender (*p* = 0.6). Respondents ranged from 18 to 76 years of age. By age quartiles and gender, female respondents clustered slightly closer to the median age than male respondents (Q_1 (M/F)_ = 37/38, Q_2 (M/F)_ = 48/50, and Q_3 (M/F)_ = 57/55). Most respondents (91%) had more than a high school level of education, and nearly a quarter of respondents (24%) had a graduate degree.

**Table 1:** Demographics of survey respondents.

Characteristics	N	%
Overall	809	
Gender		
male	630	78
female	156	19
do not wish to specify	23	3
Age, yr		
18–29	85	11
30–39	146	18
40–49	184	23
50–59	228	28
60–69	119	15
70+	47	6
Education		
graduate degree	192	24
college degree or college classes	534	67
high school, GED, orsome high school	75	9
Country		
United States	628	80

**Figure 1:**
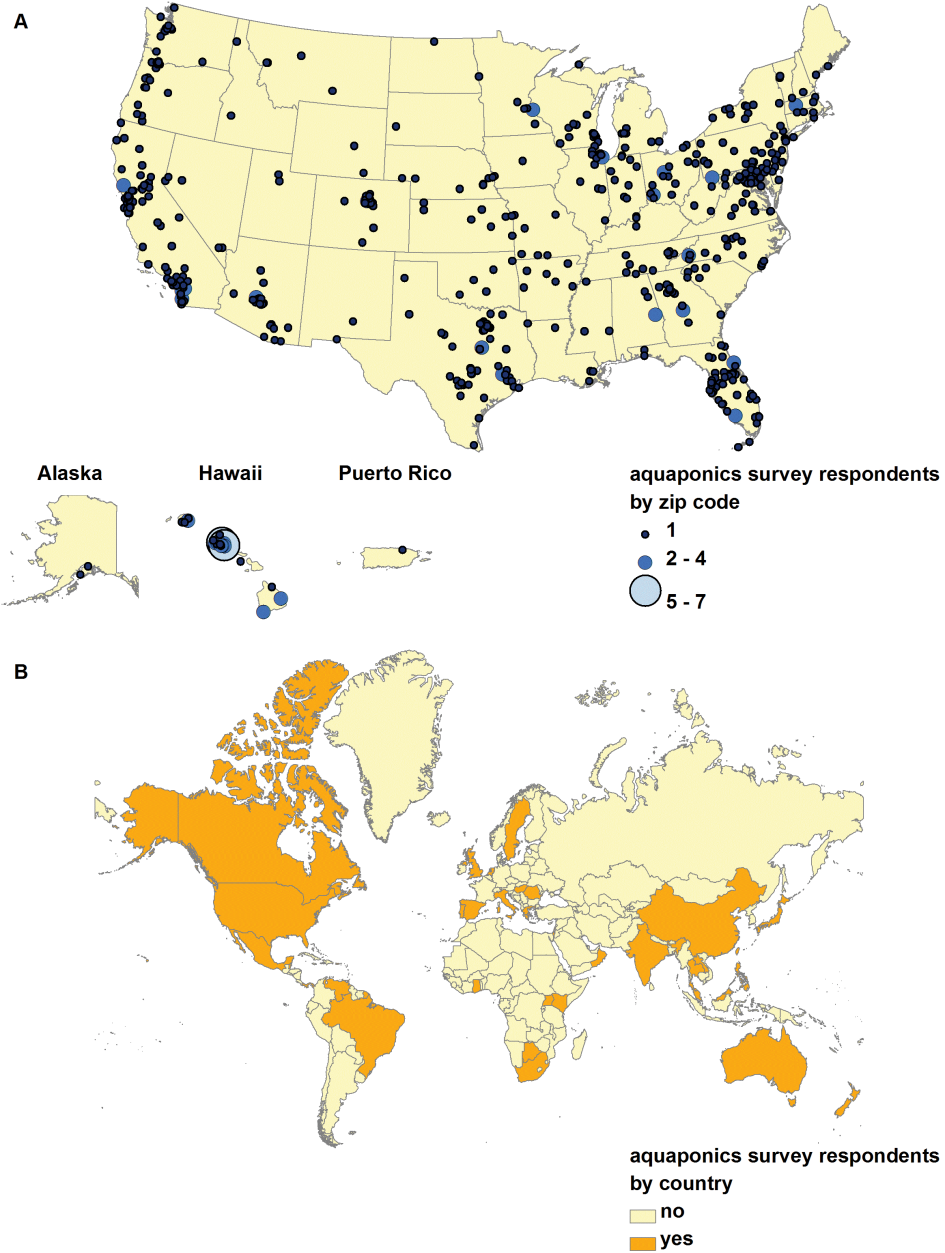
Map of survey respondents by A) United States zip code (n = 600) and B) country (n = 779 respondents from 43 countries).

The majority of respondents (80%) lived in the US, while a substantial number of respondents also lived in Australia (8%) and Canada (2%) (Figure 1). By region of the world, respondents lived in Asian and the Pacific Islands (n = 10 countries), Western and Central Europe (n = 10 countries), North, Central and South America (n = 8 countries), the Caribbean (n = 6 countries), Africa (n = 6 countries), and the Middle East (n = 2 countries).

### Background and experiences

Respondents were asked a series of yes/no questions about the aquaponics-related activities they were involved in over the previous 12 months, and these responses were used to identify three overlapping populations within the study sample (Figure S1). Of all survey respondents, 84% (n = 677) reported involvement in aquaponics as a hobby (*i.e.*, not as their primary occupation), 57% (n = 462) performed educational activities using aquaponics, and 32% (n = 257) were engaged in the commercial sale of aquaponic crops, fish materials, or services. Respondents often participated in a combination of hobbyist, educator, and commercial activities. For example, slightly more than half (51%) of hobbyists also were aquaponic educators (such as giving tours of their operation), and a quarter of hobbyists sold aquaponic products or services. After excluding hobbyists and commercial operations that engage in education, the remaining educators were from primary or secondary schools (n = 36), colleges or universities (n = 53), and vocational or technical schools (n = 11). Respondents that engaged in any commercial activities included those individuals who sold crops or fish (n = 95), materials or services (n = 69), or both (n = 93).

**Figure 2:**
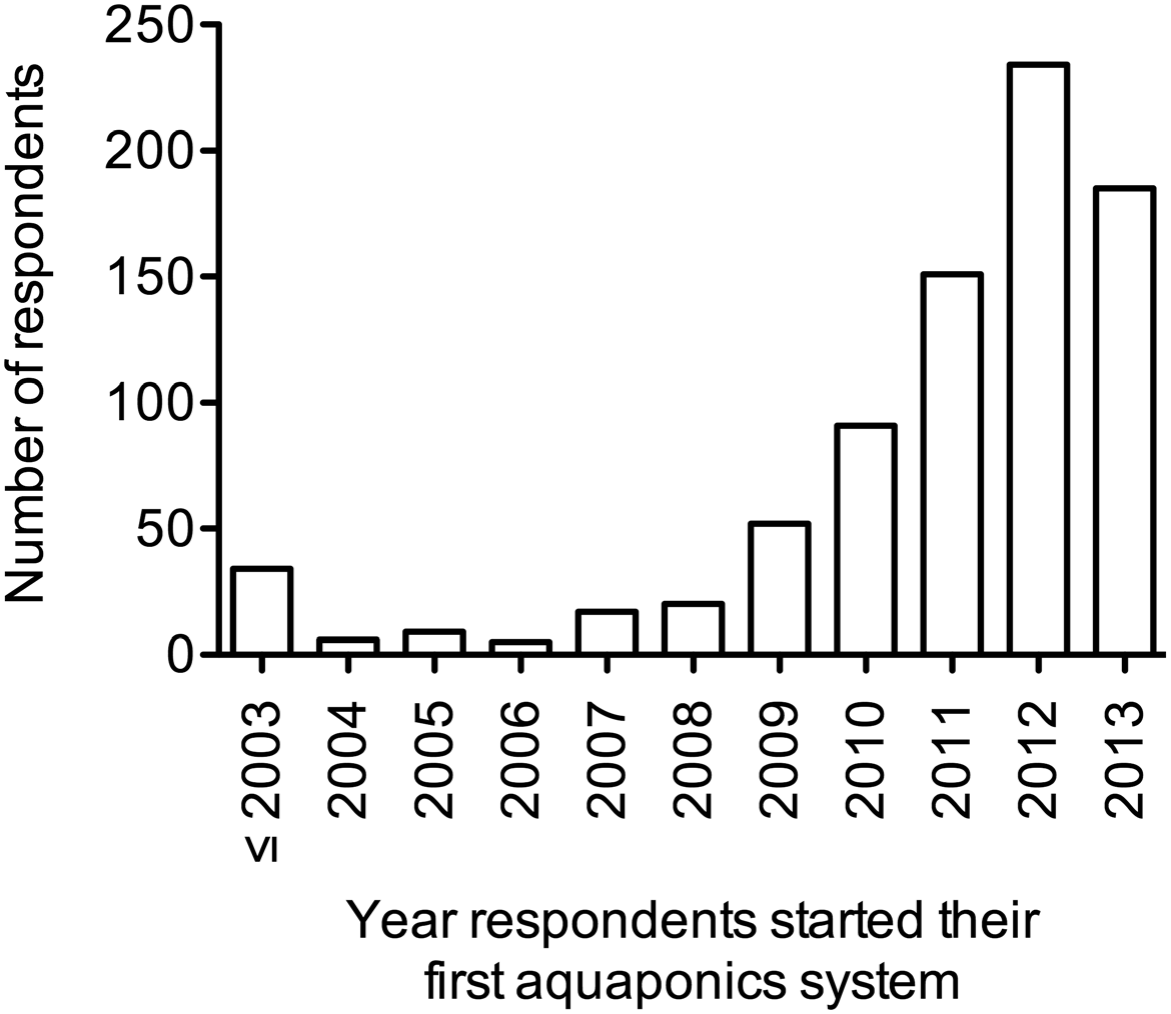
Year respondents started their first aquaponic systems (n = 804).

Respondents were asked to report the year they started their first aquaponic system (Figure 2). Nearly nine in ten respondents (89%) had ≤5 years experience with aquaponics, and over half of respondents (52%) had ≤3 years experience with aquaponics. By decade, the first aquaponic system was built by a respondent in 1974, four systems were built in the 1980s, 17 in the 1990s, 121 in the 2000s, and 661 systems were built from 2010 to 2013.

### Facility size, location, and design

**Figure 3:**
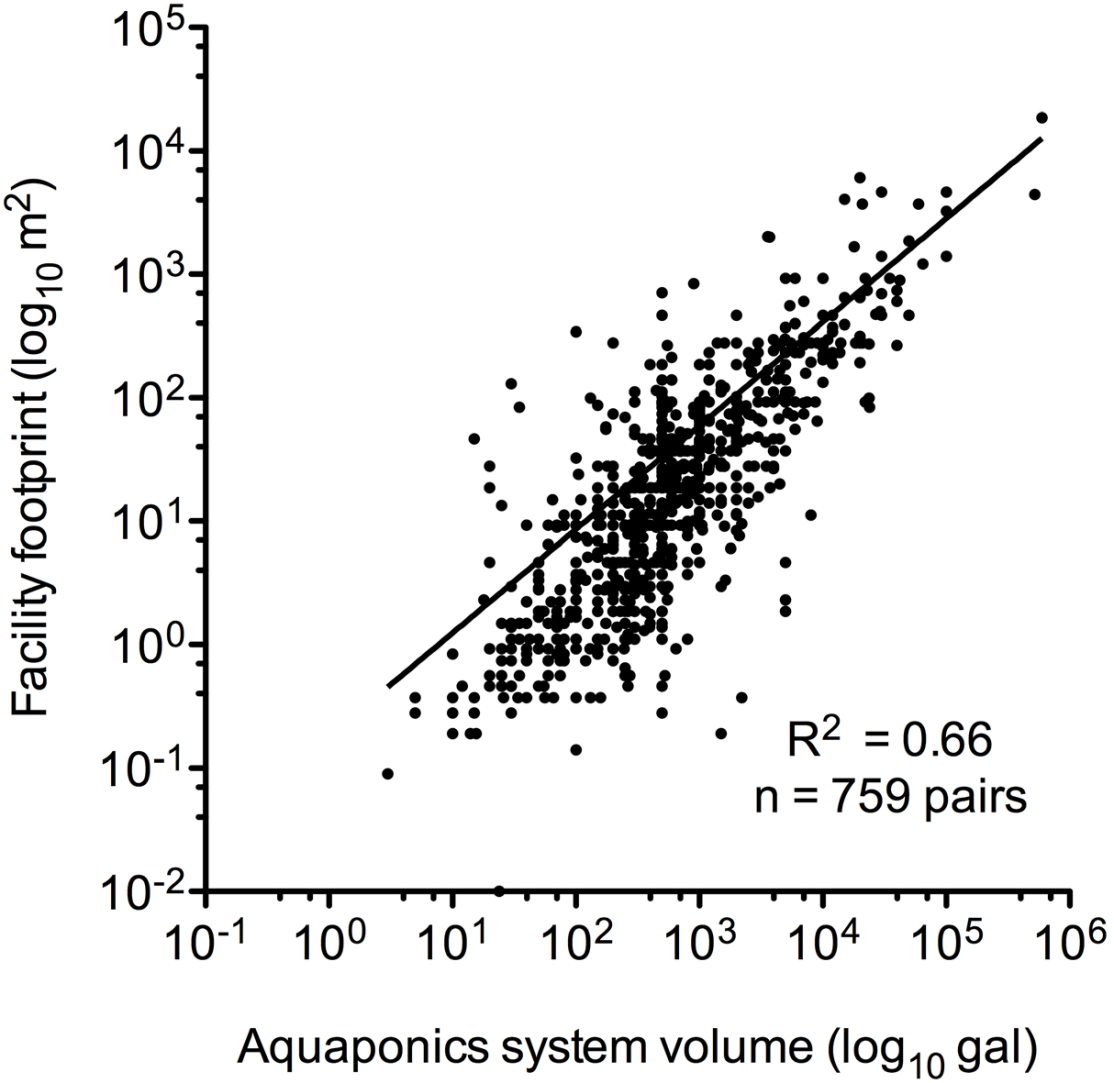
Correlation between the facility footprint size and the water volume contained within survey respondents’ aquaponic systems.

The aquaponic systems in this survey varied widely in size (Figure 3). The sum of all respondents’ aquaponic system volumes was 3.5 million gal of water, which was housed in facilities totaling 11 hectares (or 28 acres). The volumes for individual aquaponic systems ranged from 3 gal to 600,000 gal and by quartiles were: Q_1_ = 200 gal, Q_2_ = 500 gal, Q_3_ = 1,425 gal. The facility footprints ranged in size from 0.01 m^2^ to 18,580 m^2^ and by quartiles were: Q_1_ = 3 m^2^, Q_2_ = 15 m^2^, Q_3_ = 61 m^2^. The volume of aquaponic system explained two-thirds of the variability (R^2^ = 0.66) in the facility size.

Respondents maintained aquaponic systems in a variety of locations, and some respondents had more than one aquaponic system per site or an aquaponic system that was spread over several locations on the site. Forty-seven percent of aquaponic systems were housed outdoors, 46% were in greenhouses or high tunnels, 28% were inside buildings, and 3% were on rooftops. Sixty percent of respondents kept their aquaponic systems on their own properties.

Eighty-three percent of aquaponic systems (n = 657) were self-designed by the respondent. The remaining 17% of respondents (n = 135) reported hiring a consultant to design the aquaponic system and/or purchasing an aquaponic kit.

**Figure 4:**
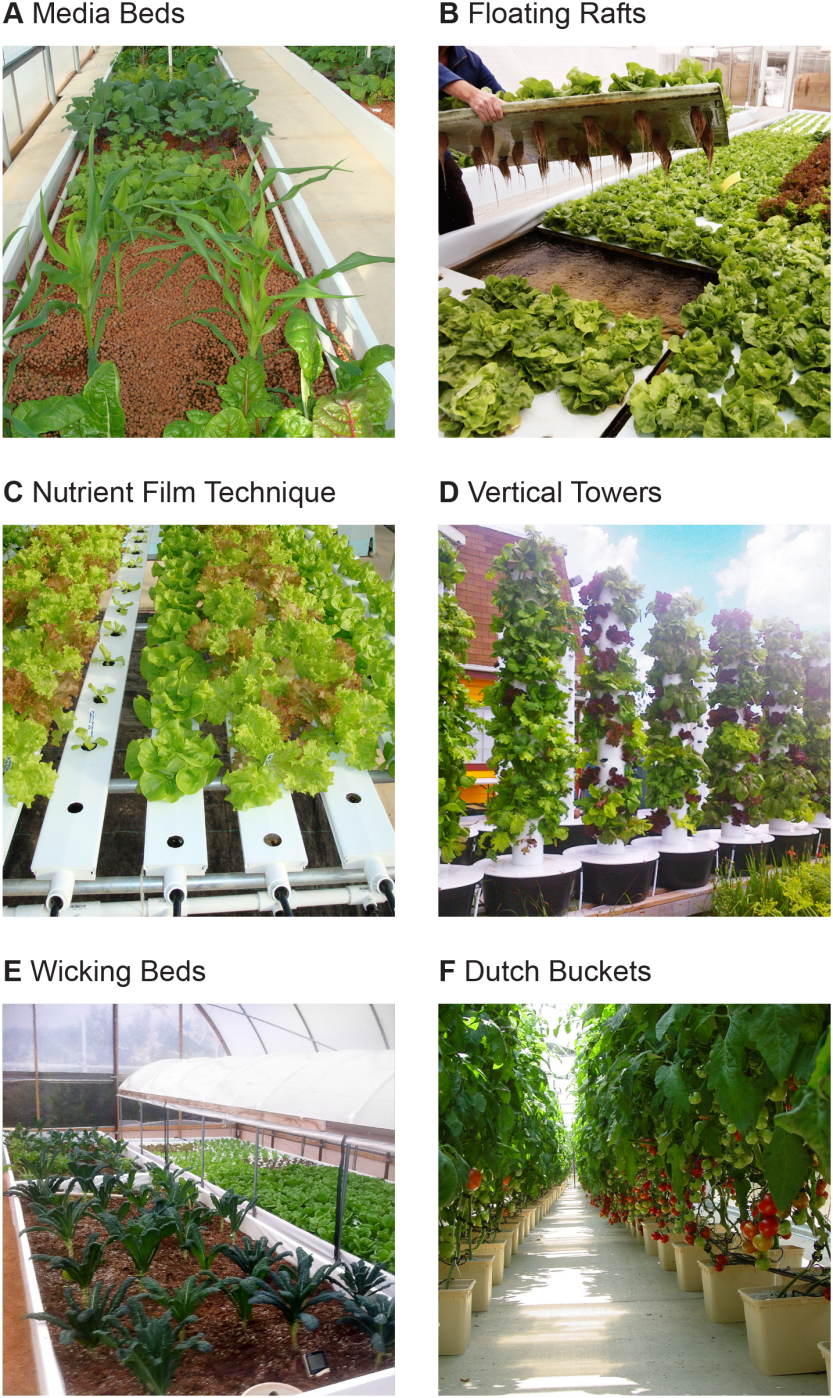
Methods for raising crops in aquaponics. Photos courtesy of Rebecca Nelson, Nelson and Pade, Inc. (A, B, C, F), Marianne Cufone, Recirculating Farms Coalition (D), and Rob Nash, Austin Aquaponics (E).

Respondents used a variety of methods for raising crops (Figure 4). The most common were containers filled with media (*i.e.,* media beds), used by 86% of respondents. Forty-six percent of respondents grew plants on floating rafts, 19% used a nutrient film technique (NFT), 17% use vertical growing towers, 2% used wicking beds, and 2% used traditional irrigation or Dutch buckets. Respondents often combined multiple growing methods; 32% of respondents used two methods to raise crops and 17% of respondents used three or more methods. The most common combination of methods used by respondents were media beds and rafts (35% of total), media beds and NFT (16%), media beds and vertical towers (13%), floating rafts and NFT (13%), and floating rafts and vertical towers (10%).

### Inputs

Water, energy, and fish feed are the three largest physical inputs for aquaponic systems. Traditional drinking water sources (*i.e.*, community piped water or well water) were the most popular types of water, used by 90% of respondents. Thirty-nine percent of respondents who use traditional drinking water sources supplemented it with rainwater capture. Surface water (*i.e.*, from streams, lakes, springs, or reservoirs) was used by 8% of respondents and mainly to supplement supply when they had no access to community piped water or well water. Untreated surface water is generally considered an unsuitable water source for aquaponics because it may contain fish and human microbial pathogens, or other organisms.

Aquaponic systems often require several mechanical devices (i.e., pumps, heaters, blowers) that use energy to operate. Electricity from the power grid was by far the most common source of energy, used by 95% of respondents. About 5% of respondents used propane or natural gas to supplement electricity from the power grid, but many more respondents (57%) used forms of renewable energy to supplement the electrical power grid. The most popular renewable energy source was sunlight: passive solar designs (22%) (*i.e.,* enclosures including greenhouses that capture sunlight for purposes of heating); solar photovoltaic cells (19%) (*i.e.* a device that converts sunlight into electrical energy); or solar thermal hot water heaters (7%) (*i.e.,* a device that uses sunlight to heat water). Wood or pellet burning stoves (6%), compost as a source of heat (3%), geothermal (3%), and wind energy (2%) were occasionally used. We identified 37 of 809 respondents (5%) as “off-the-grid,” meaning they were not powered by the electrical power grid. Twenty-eight off-the-grid respondents only used renewable sources of energy and nine off-the-grid respondents used a combination of renewable sources, propane, or natural gas.

To feed their fish, the vast majority of respondents (94%) use feed pellets, which are usually sold commercially as a complete feed. Some respondents supplemented the use of feed pellets with alternative sources: aquatic plants (33%); live feed (i.e., black soldier flies, earthworms) (30%); or to a lesser extent human food scraps (13%). Four respondents (0.5%) fed cat or dog food to fish. Respondents who used one type of alterative feed were more likely to also use other types of alternative feeds. For example, 55% of respondents who used aquatic plants as feed also used live feed, and 62% of respondents who used aquatic plants or live feed also used human food scraps.

### Outputs

**Figure 5:**
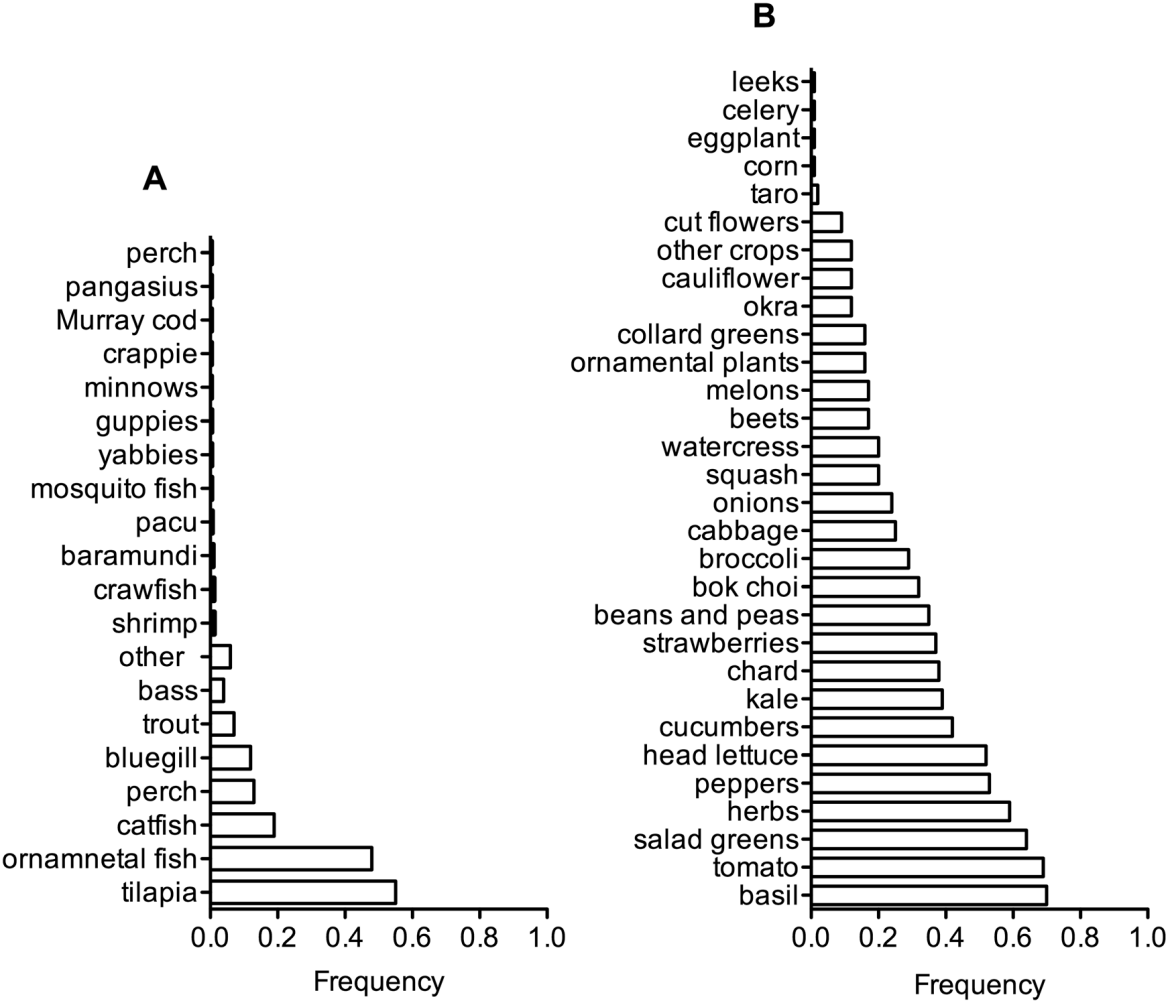
The frequency of respondents who raised A) fish and B) crops in the previous 12 months.

The most common animals raised in aquaponic systems were tilapia (55%) and ornamental fish (*i.e.,* koi, goldfish, tropical fish) (48%), with a complete list of animals raised by respondents in Figure 5a. Respondents often raised several species; 27% of respondents raised two species of fish and 18% of respondents raised three or more species of fish. There was a strong preference towards raising one or more edible species of fish (81%) compared to only raising ornamental fish (19%).

The three most common crops in aquaponic systems were basil, tomatoes, and salad greens, which were grown by 70%, 69%, and 64% of respondents in the previous 12 months (over a time period that could include June 2012 to October 2013). A complete list of crops raised by respondents is reported in Figure 5b. The average respondent grew 8±5 crops in the previous 12 months. The number of crops by a respondent ranged from 1 to 26 and by quartiles were: Q_1_ = 5 crops, Q_2_ = 8 crops, Q_3_ = 12 crops.

### Knowledge and attitudes

**Figure 6:**
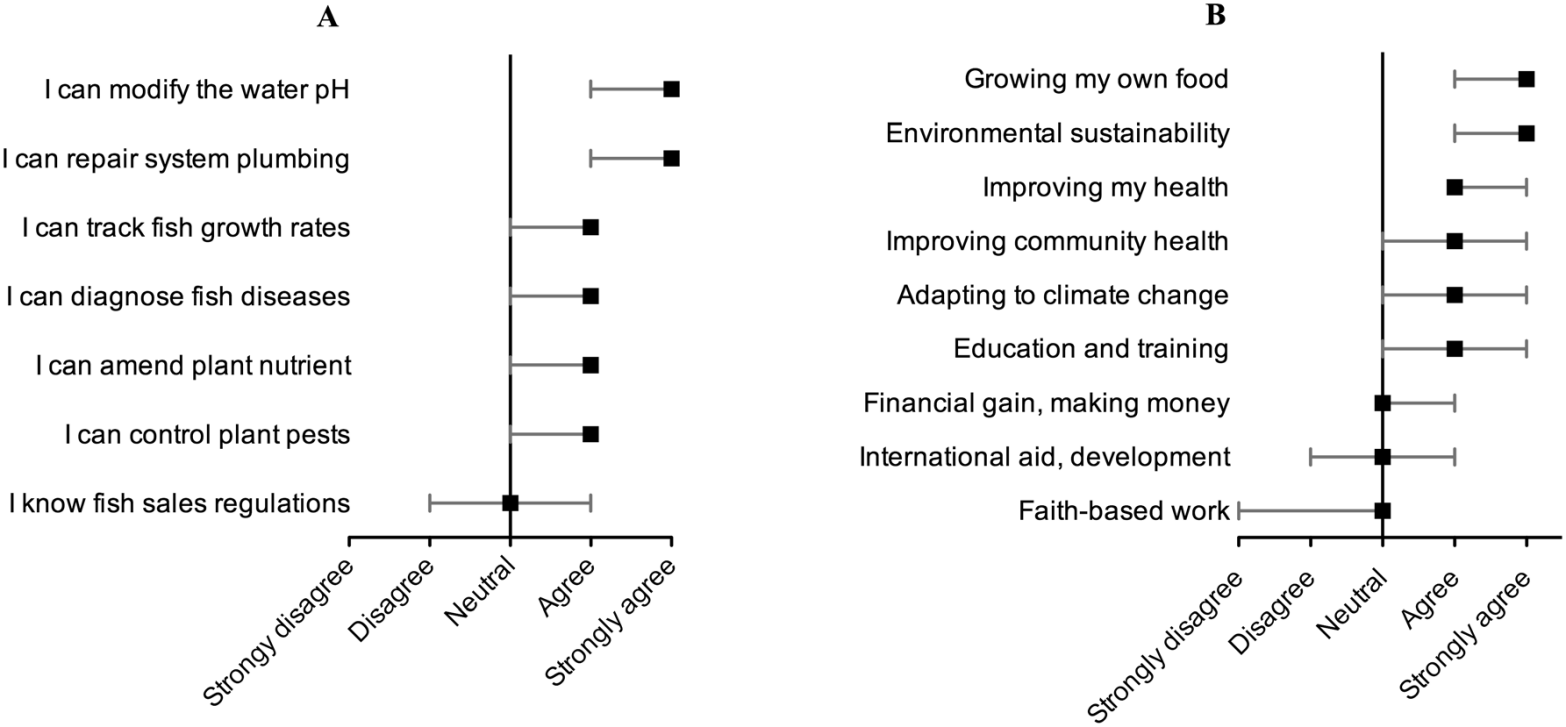
Survey respondents’ A) knowledge (n = 805) and B) personal priorities (n = 803) for his/her work in aquaponics using a Likert scale. Squares represent median values and error bars represent the interquartile range.

Respondents were asked about their knowledge regarding several topics in aquaponics including fish and plant health, maintenance of an aquaponics system, and regulations related to commercial aquaponics. The median response and interquartile range are presented in Figure 6a. Respondents strongly agreed that they knew how to amend the water pH and repair plumbing, which are key areas for maintaining a functioning aquaponic system. Respondents agreed that they had knowledge of fish and plant health, although the bottom quartile of respondents was less knowledgeable. Respondents varied in their knowledge of regulations around fish harvesting and sales, which is expected because only a sub-set of the respondents raised fish commercially in the study sample.

Respondents were asked what motivated their work in aquaponics in relation to nine issues that ranged from personal to societal issues. Respondents strongly agreed that growing their own food and environmental sustainability were priorities for their work (Figure 6b). Respondents agreed that improving their health and the health of their communities were priorities, as well as using aquaponics to adapt to climate change and using aquaponics for education and training. Faith-based work, international aid, and commercial sales were topics listed in the survey that did not motivate most respondents.

## Discussion

This study is the first large-scale survey of practitioners of aquaponics, and our findings may serve as a baseline for future research, policy, advocacy, and outreach about this growing form of agriculture. Based on survey responses, aquaponics is experiencing a period of rapid growth where participants are innovators and early adopters of technology. Aquaponics is being practiced in at least 43 countries around the world and on every continent. The majority of respondents were from the US, which may be skewed because the survey originated in the US and was not offered in other languages than English. The mean age of respondents was 47 years of age, a decade younger than the average farmer in the US [29], which may represent recruitment into farming ranks, although most respondents were not full-time farmers. Gender parity was not observed among respondents (78% male), and this aligns with the USDA Agriculture Census data showing 86% of US farmers are male [29]. Most respondents were practicing aquaponics as a hobby, had three years or less of experience with aquaponics, and were knowledgeable about maintaining their own system infrastructure, fish, and crops.

In addition to hobbyists, there were several other groups of respondents, including: educators who practice aquaponics in primary and secondary schools, vocational training centers, colleges, and universities; non-profit organizations that operate aquaponic systems; and commercial operators and consultants that sell goods, materials, and services. Analyzing the survey data by group was outside the scope of this manuscript.

Aquaponic systems ranged in size over five orders of magnitude, from indoor countertop systems to the largest commercial system built on 1.9 hectarces (4.6 acres) of land. The average aquaponic system was designed by the respondent and housed on his/her property either indoors or in a greenhouse. The average system contained 500 gallons of water and took up 15 m^2^ of space. These findings indicate that, currently, aquaponics is primarily a niche or “backyard” activity, but the methods are highly scalable to commercial systems if the basic principles and ratios of fish stocking density, feeding rates, and crop growing area are maintained [20].

There has been some debate about the best approach for raising crops in aquaponic systems. Published comparisons of crop production methods are rare, although one study found lettuce grew best by the following order of methods: media beds > floating raft > nutrient film technique [30], which aligns with the frequency of crop production methods reported by respondents in this study. In this survey, the most common method for raising crops was a media bed, however optimal crop methods may vary by the scale of the operation. Our findings indicate that experimentation in crop production is active and ongoing; almost a third of respondents used two or more methods to raise crops, and a total of seven methods were used by respondents. Continued research, optimization, and communication of the best crop production methods are needed among the aquaponics community.

Aside from labor, the major inputs for aquaponics facilities are water, energy, and fish feed. We found that respondents primarily filled their systems using community piped water or well water, ran mechanical systems using electricity from the power grid, and fed animals a commercial pelletized fish feed. Respondents were open to supplementing conventional water, energy, and feed sources with sustainable alternatives. Thirty-nine percent of respondents used rainwater capture to supplement water use, 57% of respondents used a form of renewable energy to supplement electricity from the grid, and 50% of respondents used some form of alternative feed (primarily live feed or aquatic plants) to supplement fish feed pellets. These findings are consistent with respondents’ attitudes; the average respondent strongly agreed that environmental sustainability was a personal priority for his/her work. To enable respondents to make better-informed decisions about inputs, studies are needed to compare fish growth rates and crop yields using conventional and alternative fish feeds. Studies are also needed of the economics of using renewable versus non-renewable energy sources. From a policy perspective, agricultural or energy policies that promote renewable energy use may find traction among aquaponic operators.

Respondents raised edible crops, with leafy greens, herbs, and tomatoes reported as the most popular. The average respondent strongly agreed that growing his/her own food was a personal priority. Tilapia, ornamental fish, and catfish were the most common animals raised by respondents. Tilapia are a model species used by many in the aquaponics community because they have the advantage of being able to survive in poor water quality, handle well, and can grow to high density in confinement [31]. Tilapia are also an omnivorous fish species, which can be viewed as an advantage for environmental sustainability. A common protein source in fish feed is fishmeal made from small pelagic fish like herring or sardines [32], which has measurable environmental, social, and economic costs [33]–[35]. Other fields of aquaculture are attempting to reduce or eliminate fishmeal and fish oil from feed [36], [37]. Aquaponic operators should continue reducing the use of fishmeal and fish oil as well, which is easier in fish species that are herbivorous or omnivorous. There are some concerns with using tiliapia; they are an invasive species with controlled use in many US states and banned in some countries (*i.e.,* Australia) [38], [39]. The narrow focus on tilapia by aquaponic researchers means that production methods have not been optimized for many other aquatic livestock. There were a wide variety of fish and crustaceans reportedly grown by respondents, and additional research is warranted on production of these species.

Limitations of this study include a lack of previously validated survey instruments available for aquaponics, and a study population that has not been well characterized, which prevents administering a survey to a random sample of individuals who practice aquaponics. Instead, the authors used a chain sampling approach and social media to identify potential participants. Due to these constraints, we could not calculate a survey response rate, and there is limited generalizability to aquaponics practitioners beyond those who responded to the study.

Three types of aquaponics producers were identified in the survey (commercial producers, hobbyists, and educators) that deserve further exploration, and in future analyses we will focus on factors that influence profitability of commercial operations, consumption of aquaponically-grown produce and fish among hobbyists, and how educators use aquaponics in their classrooms. The results of this survey can be compared to future qualitative and quantitative studies of aquaponics producers to confirm or refine our findings and to track trends in the field. In addition to more research, outreach and communication efforts are needed to translate findings to individuals engaged in aquaponics, and to elicit feedback about future directions of study and important policy issues.

## Conclusions

These survey results expand our understanding of aquaponics producers and their demographics, motivations, and production systems. Aquaponics producers have a large and active community. Most survey participants were hobbyists, however, a significant proportion of respondents were educators, staff of non-profit organizations, or commercial producers. Primary reasons respondents cited for their engagement in aquaponics were to grow their own food, advance environmental sustainability, and improve personal health. Aquaponics operations vary in size and type of production system, and we found a high adoption rate among respondents towards environmentally sustainable methods of production. These findings can help inform aquaponics practices and policy decisions, and serve as a baseline for exploring future trends in aquaponics.

## Supporting Information

Appendix S1 Contains the survey codebook used in this study.
(PDF)

Figure S1 Venn diagram of respondents’ backgrounds and experiences in aquaponics in the previous 12 months. The survey was open from June to October 2013. The Venn diagram was constructing using software eulerAPE v.3 [1], and population sample size is reported inside the ovals.
(PDF)
