# Supplementary material for: An International Survey of Aquaponics Practitioners
Source: PLoS One. 2014 Jul 16;9(7):e102662. doi: 10.1371/journal.pone.0102662 (PMC4100909; doi:10.1371/journal.pone.0102662)
Supplement: Appendix S1 — Contains the survey codebook used in this study. (PDF) [file pone.0102662.s001.pdf]

Supporting Information

Appendix S1. Aquaponics Survey Codebook

Journal: PLOS ONE

Title: An International Survey of Aquaponics Practitioners

Short title: International Survey of Aquaponics

Authors: David C. Love [1,2], Jillian P. Fry [1,2], Laura Genello [1,2], Elizabeth S. Hill [3], J. Adam Frederick [4] Ximin Li [5], Ken Semmens [6]

Affiliation:

[1] Johns Hopkins Center for a Livable Future, Johns Hopkins University, Baltimore, Maryland, USA

[2] Department of Environmental Health Sciences, Bloomberg School of Public Health, Johns Hopkins University, Baltimore, Maryland, USA

[3] University of Maryland Extension, Baltimore, Maryland USA

[4] Maryland Sea Grant College, University of Maryland College Park, Maryland, USA

[5] Department of Biostatistics, Bloomberg School of Public Health, Johns Hopkins University, Baltimore, Maryland, USA

[6] Agriculture and Natural Resources Unit, West Virginia University Extension Service, Morgantown, West Virginia, USA

consent Would you like to take the survey?

- Yes (1)
- Not now, email me the survey (2)
- No (3)

If “No” is selected, then skip to end of survey. If “Not now, email me the survey” is selected, then skip to email later.

emaillater Contact Information

This information will be kept confidential and will not be released with your study responses.

First Name (1)
Last Name (2)
Email (3)

This survey is intended for individuals 18 years of age or older in the United States or ANY country who are engaged in aquaponics. Aquaponics is a food production system that combines aquaculture (raising fish) with hydroponics (raising plants in soilless environment).

ever_pract Have you or has your organization EVER practiced aquaponics?

- Yes (1)
- No (2)

If No Is Selected, Then Skip To Enthusiast Questions

GENERAL INFORMATION

The following questions cover general information about your aquaponics operation.

orgcat My survey responses will reflect WHICH of the following perspectives? SELECT ONE

- ❍ my PERSONAL activities with aquaponics (1)
- ❍ the activities of my ORGANIZATION or COMPANY (2)

prev12op In the previous 12 months, did you or your organization OPERATE and MAINTAIN an aquaponics system?

- ❍ Yes (1)
- ❍ No (2)

Display if prev12op=1

hobby In the previous 12 months, did you practice aquaponics as a HOBBY (not as your primary occupation)?

- ❍ Yes (1)
- ❍ No (2)

Display if prev12op=1

sell_crops In the previous 12 months, did you or your organization SELL your aquaponics-raised FISH or CROPS?

- ❍ Yes (1)
- ❍ No (2)

Display if prev12op=1

sell_mater In the previous 12 months, did you or your organization SELL aquaponics-related MATERIALS, SERVICES, or INFORMATION (e.g. consulting, design, equipment sales)?

- ❍ Yes (1)
- ❍ No (2)

educate In the previous 12 months, did you or your organization EDUCATE people about aquaponics (e.g. host tours, classes, courses, lectures, or workshops)?

- ❍ Yes (1)
- ❍ No (2)

Display if educate=1

edu_cat Which of the following categories BEST describes your organization? SELECT THE BEST CHOICE

- ❍ K-12 Primary and Secondary School (1)
- ❍ 2-year or 4-year College or University or Graduate School (2)
- ❍ Vocational or Trade School or Technical Training Center (3)
- ❍ Commercial Producer, Vendor, or Consultant (4)
- ❍ Non-Profit Organization (e.g. after school program, faith-based organization, international aid organization, workforce training organization) (5)
- ❍ Other (88) ____________________

  - many listed “other” and said “hobbyist/individual”

FACILITY INFORMATION

Display if prev12op=1

The following questions cover aspects of your aquaponics system and facility.All responses will be kept confidential, and no personal or professional identifiable information will be released with reports using these data.

first What year did you start your FIRST aquaponics system?

Year (XXXX) (1)

locate Where is your aquaponics system housed? CHECK ALL THAT APPLY.

- ☐ Inside a building (1)
- ☐ Inside a greenhouse or high tunnel (2)
- ☐ On a rooftop (3)
- ☐ Outdoors (4)
- ☐ At your home (5)

design Who designed your aquaponics system? CHECK ALL THAT APPLY.

- ☐ Self-designed (1)
- ☐ Purchased a kit (2)
- ☐ Designed by consultants (3)

size Please ESTIMATE the size of your aquaponics system. For metric users, please convert units: Liters to Gallons liquid conversion calculatorMeters to Feet length conversion calculator

Water volume in the aquaponics system (in gallons) (1)__________________
Footprint of facility (in square feet) (2) _______________

water What WATER SOURCES were used in the aquaponics system in the previous 12 months? CHECK ALL THAT APPLY.

- ❑ Municipal or community piped water (1)
- ❑ Groundwater or well water (2)
- ❑ Surface water (from streams, lakes, springs, or reservoirs) (3)
- ❑ Rainwater (4)
- ❑ Other (88) ____________________

energy What ENERGY SOURCES were used to operate the aquaponics system in the previous 12 months? CHECK ALL THAT APPLY.

- ❑ Electricity from the power grid (1)
- ❑ Natural gas (2)
- ❑ Propane (3)
- ❑ Compost (as a source of heat) (4)
- ❑ Geothermal (5)
- ❑ Passive solar (such as a greenhouse) (6)
- ❑ Solar thermal hot water (7)
- ❑ Solar photovoltaic cells (8)
- ❑ Wind energy (9)
- ❑ Wood-burning / pellet stove or boiler (10)
- ❑ Other (88) ____________________

Display if sell_crops=1 OR sell_mater=1

employ How many PEOPLE (including yourself) were EMPLOYED at your organization in the previous 12 months?

	Number of workers (1)
Full-time workers (150 days or more per year) (1)	____________________
Part-time workers (less than 150 days per year) (2)	____________________
Unpaid workers, family members, volunteers (3)	____________________

Display if sell_crops=1

facilities Please answer the following questions about your organization:

	Yes (1)	No (2)	Not applicable (98)
Do you or your organization own the property where the aquaponics operation is housed? (**ownproperty**)	❍	❍	❍
Is supplemental lighting used for plant production? (**supplight**)	❍	❍	❍
Is there on-site cold storage for produce? (**coldstore**)	❍	❍	❍
Is there an on-site bathroom and hand-washing station? (**onsitebath**)	❍	❍	❍
Does your organization have a food safety plan? (**foodsafeplan**)	❍	❍	❍
Does your organization have an aquaculture permit? (**agpermit**)	❍	❍	❍
Does your organization breed fish in a hatchery or nursery? (**hatchery**)	❍	❍	❍
Does your organization sell **other** agricultural products? (e.g row crops, honey, livestock, etc.) (**otherag**)	❍	❍	❍
Does your organization produce and sell any USDA organic certified **aquaponics-raised plant products**? (**USDAcert**)	❍	❍	❍
If **fish** could be certified USDA organic, would you be interested in pursuing this certification? (**wantUSDAcert**)	❍	❍	❍

FISH AND CROP PRODUCTION

Display if prev12op=1

These questions cover the types of fish and crops raised in your aquaponics system.

All responses will be kept confidential, and no personal or professional identifiable information will be released with reports using these data.

fish_type What types of FISH were raised at your aquaponics facility in the previous 12 months? CHECK ALL THAT APPLY.

- ❑ Tilapia (1)
- ❑ Catfish (2)
- ❑ Bluegill (3)
- ❑ Perch (4)
- ❑ Ornamental fish (Koi, Goldfish, Tropical fish) (5)
- ❑ Bass (6)
- ❑ Trout (7)
- ❑ Other (88) ____________________

Display if prev12op=1 AND sell_crops=1

fish_harv Please ESTIMATE your aquaponics FISH HARVEST in the previous 12 months at your facility. *For metric users, please convert units to pounds: Kilograms to Pounds conversion calculator*

- ❍ None (1)
- ❍ 1 - 49 pounds (2)
- ❍ 50 - 99 pounds (3)
- ❍ 100 - 499 pounds (4)
- ❍ 500 - 999 pounds (5)
- ❍ 1,000 - 4,999 pounds (6)
- ❍ 5,000 - 9,999 pounds (7)
- ❍ 10,000 pounds or greater (8)

crop_type What types of CROPS were raised at your aquaponics facility in the previous 12 months? CHECK ALL THAT APPLY.

- ❑ Beans and Peas (1)
- ❑ Cucumbers (2)
- ❑ Melons (3)
- ❑ Okra (4)
- ❑ Peppers (5)
- ❑ Squash (6)
- ❑ Strawberries (7)
- ❑ Tomatoes (8)
- ❑ Basil (9)
- ❑ Bok choi (10)
- ❑ Collard greens (11)
- ❑ Head lettuce (12)
- ❑ Herbs (13)
- ❑ Kale (14)
- ❑ Salad greens (15)
- ❑ Watercress (16)
- ❑ Beets (17)
- ❑ Carrots (18)
- ❑ Onions (19)
- ❑ Cut flowers (20)
- ❑ Ornamental plants (21)
- ❑ Taro (22) -- not listed but added due to overwhelming response in “other” category
- ❑ Other fruit (23) - not listed but added due to overwhelming response in “other” category
- ❑ Corn (24) - not listed but added due to overwhelming response in “other” category

- ❑ Eggplants (25)- not listed but added due to overwhelming response in “other” category
- ❑ Other rooting crops (26) - not listed but added due to overwhelming response in “other” category
- ❑ Gourds and melons (27) - not listed but added due to overwhelming response in “other” category
- ❑ Duckweed (28) - not listed but added due to overwhelming response in “other” category
- ❑ Leeks (29) not listed but added due to overwhelming response in “other” category
- ❑ Celery (30) not listed but added due to overwhelming response in “other” category
- ❑ Chard (31)
- ❑ Broccoli (32)
- ❑ Cabbage (33)
- ❑ Cauliflower (34)
- ❑ Other (88) ____________________

Display if prev12op=1 AND sell_crops=1

plant_harv Please ESTIMATE your aquaponics CROP HARVEST in the previous 12 months at your facility. *For metric users, please convert units to pounds: Kilograms to Pounds conversion calculator*

- ❍ None (1)
- ❍ 1 - 49 pounds (2)
- ❍ 50 - 99 pounds (3)
- ❍ 100 - 499 pounds (4)
- ❍ 500 - 999 pounds (5)
- ❍ 1,000 - 4,999 pounds (6)
- ❍ 5,000 - 9,999 pounds (7)
- ❍ 10,000 - 49,999 pounds (8)
- ❍ 50,000 - 99,999 pounds (9)
- ❍ 100,000 pounds or greater (10)

fish_feed What types of FISH FEED were used in the previous 12 months? CHECK ALL THAT APPLY.

- ❑ Feed pellets (dry fish feed) (1)
- ❑ Aquatic plants (duckweed, azolla, etc.) (2)
- ❑ Live feed (insect larvae, black soldier flies, earthworms, etc.) (3)
- ❑ Food scraps (4)
- ❑ Other (88) ____________________

crop_meth Which of the following CROP PRODUCTION METHODS were used in the previous 12 months? CHECK ALL THAT APPLY.

- ❑ Rafts (planting in floating trays) (1)
- ❑ Media Beds (gravel or other media filled boxes) (2)
- ❑ Nutrient Thin Film (horizontal irrigated trays or gutter planters) (3)
- ❑ Vertical Towers (vertical irrigated pipe planters) (4)
- ❑ Wicking Beds (5) – not part of the survey but added due to the high response rate in "other"
- ❑ Irrigation and Dutch Buckets (6) – not part of the survey but added due to the high response rate in "other"
- ❑ Other (88) ____________________

EDUCATIONAL ACTIVITIES USING AQUAPONICS

Display if educate=1

The following questions are specific to activities around aquaponics and education. All responses will be kept confidential, and no personal or professional identifiable information will be released with reports using these data.

tours In the past 12 months, ESTIMATE how many individuals have taken part in TOURS, CLASSES, COURSES, or WORKSHOPS at your aquaponics facility or classroom?

- ❍ None (1)
- ❍ 1 - 24 (2)
- ❍ 25 - 49 (3)
- ❍ 50 - 99 (4)
- ❍ 100 - 499 (5)
- ❍ 500 - 999 (6)
- ❍ 1,000 - 4,999 (7)
- ❍ 5,000 or greater (8)

edusubject What SUBJECTS do you TEACH that incorporate aquaponics? CHECK ALL THAT APPLY.

- ❑ Agriculture (1)
- ❑ Aquaculture (2)
- ❑ Biology (3)
- ❑ Chemistry (4)
- ❑ Earth Science (5)
- ❑ Engineering (6)
- ❑ Environmental Science (7)
- ❑ Food Systems (8)
- ❑ Horticulture (9)
- ❑ Math (10)
- ❑ Marine Science (11)
- ❑ Physics (12)
- ❑ Public Health (13)
- ❑ Vocational Technology (14)
- ❑ Aquaponics (as a stand-alone subject) (15)
- ❑ Permaculture (16) – not part of the survey but added due to the high response rate in
- ❑ Hydroponics (17) – not part of the survey but added due to the high response rate in
- ❑ Sustainability (18) – not part of the survey but added due to the high response rate in
- ❑ Other (88) ____________________

edu_target Which GROUPS do you target in your education or outreach activities? CHECK ALL THAT APPLY.

- ❑ 5th grade and younger (1)
- ❑ 6th - 8th grade (2)
- ❑ 9th - 10th grade (3)
- ❑ 11th - 12th grade (4)
- ❑ Vocational or trade school or training center (5)
- ❑ 2-year college or community college (6)
- ❑ 4-year college or university (7)
- ❑ Graduate school (8)
- ❑ Working-age adults (9)
- ❑ Retired adults (10)
- ❑ All ages (11)

Display if edu_cat=1, 2 or 3

edu_matrix Please answer the following questions about aquaponics in your EDUCATION SYSTEM.

	Yes (1)	No (2)	Not Applicable (98)
Is aquaponics integrated in your curriculum? (1)<br><br>Are there courses where aquaponics is used for instruction? (2)	❍	❍	❍
Do your students have opportunities to conduct self-guided aquaponics research projects? (3)	❍	❍	❍
Does aquaponics have support from the leadership of your organization? (4)	❍	❍	❍
	❍	❍	❍

AQUAPONICS SALES AND PROFITABILITY

Display if sell_crop=1 OR sell_mater=1

The following questions are specific to organizations or individuals that sell agricultural products.

All responses will be kept confidential, and no personal or professional identifiable information will be released with reports using these data.

Display if sell_crops=1

outlets To which OUTLETS did your organization sell aquaponics-raised food to in the previous 12 months? CHECK ALL THAT APPLY.

	Crops (2)	Fish (1)
At your farm or facility (**farmo**)	❑	❑
Road-side stand (**roadsideo**)	❑	❑
Farmers market (**fmarketo)**	❑	❑
Community supported agriculture (CSA) **(CSAo)**	❑	❑
Restaurants (**restauranto**)	❑	❑
Grocery stores (**groceryo**)	❑	❑
Institutional buyers (schools, hospitals, corporations, etc.) (**instituteo**)	❑	❑
Wholesale distributor, live hauler, brokers (**wholesaleo**)	❑	❑
To other producers (sale of juvenile fish, plant starts, etc.) (**otherproducero**)	❑	❑
Other (**88outlet**)	❑	❑

personinc Was an aquaponics-related job your PRIMARY source of income in the PREVIOUS 12 months?

- ❍ Yes (1)
- ❍ No (2)
- ❍ Prefer not to disclose (98)

profitable Please respond to the following questions about the economic sustainability of your business:

	Yes (1)	No (2)	Prefer not to disclose (98)
Was the aquaponics portion of your business PROFITABLE in the PREVIOUS 12 months? (**prev12profit**)	❍	❍	❍
Will the aquaponics portion of your business be PROFITABLE in the NEXT 12 months? **(next12profit**)	❍	❍	❍
Will the aquaponics portion of your business be PROFITABLE in the NEXT 36 months (3 years)? (**next36profit**)	❍	❍	❍

sales Please ESTIMATE your or your organization's AQUAPONICS-RELATED GROSS SALES REVENUE in the previous 12 months? (Gross sales revenue includes the sale of plants, fish, consulting fees, equipment sales, speaking fees, etc.) For international respondents, please convert units to US Dollars: Currency Conversion Calculator

- ❍ None (1)
- ❍ $1 - $499 USD (2)
- ❍ $500 - $999 USD (3)
- ❍ $1,000 - $4,999 USD (4)
- ❍ $5,000 - $9,999 USD (5)
- ❍ $10,000 - $49,999 USD (6)
- ❍ $50,000 - $99,999 USD (7)
- ❍ $100,000 - $499,999 USD (8)
- ❍ $500,000 USD or greater (9)
- ❍ Prefer not to disclose (98)

FUNDING AND INVESTMENTS IN AQUAPONICS

Display if prev12op=1 OR sell_mater=1 OR educate=1

These questions address the financial inputs into your aquaponics facility. All responses will be kept confidential, and no personal or professional identifiable information will be released with reports using these data.

fundsource What SOURCES of money supported your aquaponics operation in the previous 12 months? CHECK ALL THAT APPLY

- ❑ Income from fish or crop sales (1)
- ❑ Income from aquaponics supplies, equipment, or materials sales (2)
- ❑ Income from aquaponics workshops, classes, tours, or public speaking fees (3)
- ❑ Income from aquaponics consulting, design, or construction fees (4)
- ❑ Government funding or grants (federal, state, or local) (5)
- ❑ Non-governmental organization gifts and grants (6)
- ❑ Private investments (7)
- ❑ Bank loans (8)
- ❑ Personal investments (9)
- ❑ Other (88) ____________________
- ❑ Prefer not to disclose (98)

invest Please ESTIMATE your or your organization's AQUAPONICS-RELATED INVESTMENTS in the previous 12 months. (investments in equipment, supplies, material or other expenses related to the aquaponics system) *For international respondents, please convert units to US Dollars: Currency Conversion Calculator*

- ❍ None (1)
- ❍ $1 - $499 USD (2)
- ❍ $500 - $999 USD (3)
- ❍ $1,000 - $4,999 USD (4)
- ❍ $5,000 - $9,999 USD (5)
- ❍ $10,000 - $49,999 USD (6)
- ❍ $50,000 - $99,999 USD (7)
- ❍ $100,000 - $499,999 USD (8)
- ❍ $500,000 USD or greater (9)
- ❍ Prefer not to disclose (98)

PERSONAL EXPERIENCE

The following questions address your experience and motivations for practicing aquaponics, and your relationship with the larger aquaponics community.All responses will be kept confidential, and no personal or professional identifiable information will be released with reports using these data.

Display if prev12op=1

eat_self On AVERAGE, how often did you or your family eat products YOU RAISED IN AN AQUAPONICS SYSTEM in the previous 12 months?

	Daily (1)	2-3 Times a Week (2)	Once a Week (3)	2-3 Times a Month (4)	Once a Month (5)	Less than Once a Month (6)	Never (7)
Fruits and Vegetables (1)	❍	❍	❍	❍	❍	❍	❍
Fish (2)	❍	❍	❍	❍	❍	❍	❍

ideology Are the following topics PERSONAL PRIORITIES for your work in aquaponics?

	Strongly Disagree (1)	Disagree (2)	Neither Agree nor Disagree (3)	Agree (4)	Strongly Agree (5)
Growing my own food (**growfood**)	❍	❍	❍	❍	❍
Improving my health (**improdiet**)	❍	❍	❍	❍	❍
Improving the health of my community (**imprcomm**)	❍	❍	❍	❍	❍
Education and training (**education**)	❍	❍	❍	❍	❍
Financial gain, making money (**money**)	❍	❍	❍	❍	❍
Faith-based work (**faith**)	❍	❍	❍	❍	❍
International aid and development (**aid**)	❍	❍	❍	❍	❍
Environmental sustainability (**env**)	❍	❍	❍	❍	❍
Adapting to climate change (**climate**)	❍	❍	❍	❍	❍

knowledge Do you agree or disagree with the following statements:

	Strongly Disagree (1)	Disagree (2)	Neither Agree nor Disagree (3)	Agree (4)	Strongly Agree (5)
I know how to modify the pH of an aquaponics system (**knowpH**)	❍	❍	❍	❍	❍
I know how to diagnose FISH diseases (**knowfishd**)	❍	❍	❍	❍	❍
I know how to diagnose PLANT nutrient deficiencies (**knowplantnut**)	❍	❍	❍	❍	❍
I know how to repair plumbing if something breaks (**knowplumb**)	❍	❍	❍	❍	❍
I know how to track FISH growth rates (**knowfishgrow**)	❍	❍	❍	❍	❍
I know how to manage PLANT pests effectively (**knowplantpests**)	❍	❍	❍	❍	❍
I know the regulations for processing and selling FISH (**knowsellfishreg**)	❍	❍	❍	❍	❍

resources What RESOURCES do you use to troubleshoot or solve problems? CHECK ALL THAT APPLY.

- ❑ Contact other growers (i.e., aquaponics, hydroponics, or traditional farmers) (1)
- ❑ Online websites, YouTube, internet community forums (2)
- ❑ Print resources (books, magazines, other publications) (3)
- ❑ Seminars or workshops (4)
- ❑ University staff / agriculture extension (5)
- ❑ State government agencies (i.e., state fisheries or natural resources agencies) (6)
- ❑ Federal government agencies (USDA, NOAA, etc) (7)
- ❑ Other (88) ____________________

DEMOGRAPHIC INFORMATION

All responses will be kept confidential, and no personal or professional identifiable information will be released with reports using these data.

Display if orgcat=2

orglocate Where is your aquaponics system located?

City (3)
State, Province, or District (4)
Zip or Postal code (5)
Country (6)
Name of Organization (7)

Display if orgcat=1

location Where is your aquaponics system located?

City (1)
State, Province or District (2)
Zip or Postal Code (3)
Country (4)

roleinorg What is your role in the organization? CHECK ALL THAT APPLY.

- ❑ Owner or Operator (1)
- ❑ CEO (2)
- ❑ Executive Director (3)
- ❑ School Official (4)
- ❑ Farm Manager (5)
- ❑ Educator (6)
- ❑ Employee (7)
- ❑ Consultant (8)
- ❑ Volunteer (9)
- ❑ Other (88) ____________________

gender What is your gender?

- ❍ Male (1)
- ❍ Female (2)
- ❍ Do not wish to specify (98)

edu_level What is your highest level of education?

- ❍ Less than High School (1)
- ❍ High School or GED (2)
- ❍ Some college (3)
- ❍ College degree (e.g.: BA, BS) (4)
- ❍ Masters degree (e.g.: MBA, MAT, MPH) (5)
- ❍ Doctorate degree (e.g.: PhD, JD, MD, DVM) (6)

age What is your age in years? _______________

finding Would you like to be contacted when the survey findings are released?

- ❍ Yes (1)
- ❍ No (2)

next_study May we contact you by e-mail for future studies?

- ❍ Yes (1)
- ❍ No (2)

prize Would you like to be entered into the lottery to win one of four $75 gift certificates to Amazon.com?

- ❍ Yes (1)
- ❍ No (2)

name_email Contact Information   This information will be kept confidential and will not be released with your study responses.

First name (1)
Last Name (2)
Email (3)

com_box Do you have any additional comments? ________________________________

QUESTIONS FOR ENTHUSIASTS

Display if ever_pract=2

interest What aspects of aquaponics are you MOST interested in? Check all that apply.

- ❑ Growing my own food (1)
- ❑ Environmental sustainability (2)
- ❑ Commercial production (3)
- ❑ Education and training (4)

enthage What is your age in years? ______________

enthulocat Where are you located?

City (1)
State, Province, or District (2)
Zip or Postal code (3)
Country (4)

follow-up Would you like to be contacted when the survey findings are released?

- ❍ Yes (1)
- ❍ No (2)

futurefoll May we contact you by e-mail for future studies?

- ❍ Yes (1)
- ❍ No (2)

contact Contact Information

This information will be kept confidential and will not be released with your study responses.

First Name (1)
Last Name (2)
Email (3)

comments Do you have any additional comments? _________________

We thank you for completing the survey.
Your response has been recorded. For any questions or comments about the survey please contact Dave Love (dlove@jhsph.edu).

You can help us track growth and interest in aquaponics.
Please share this survey link with your friends and colleagues:

www.jhsph.edu/clf/survey_aquaponics.html
