# Supplementary material for: An International Survey of Aquaponics Practitioners
Source: PLoS One. 2014 Jul 16;9(7):e102662. doi: 10.1371/journal.pone.0102662 (PMC4100909; doi:10.1371/journal.pone.0102662)
Supplement: Figure S1 — Venn diagram of respondents’ backgrounds and experiences in aquaponics in the previous 12 months. The survey was open from June to October 2013. The Venn diagram was constructing using software eulerAPE v.3 [1], and population sample size is reported inside the ovals. (PDF) [file pone.0102662.s002.pdf]

Supporting Information

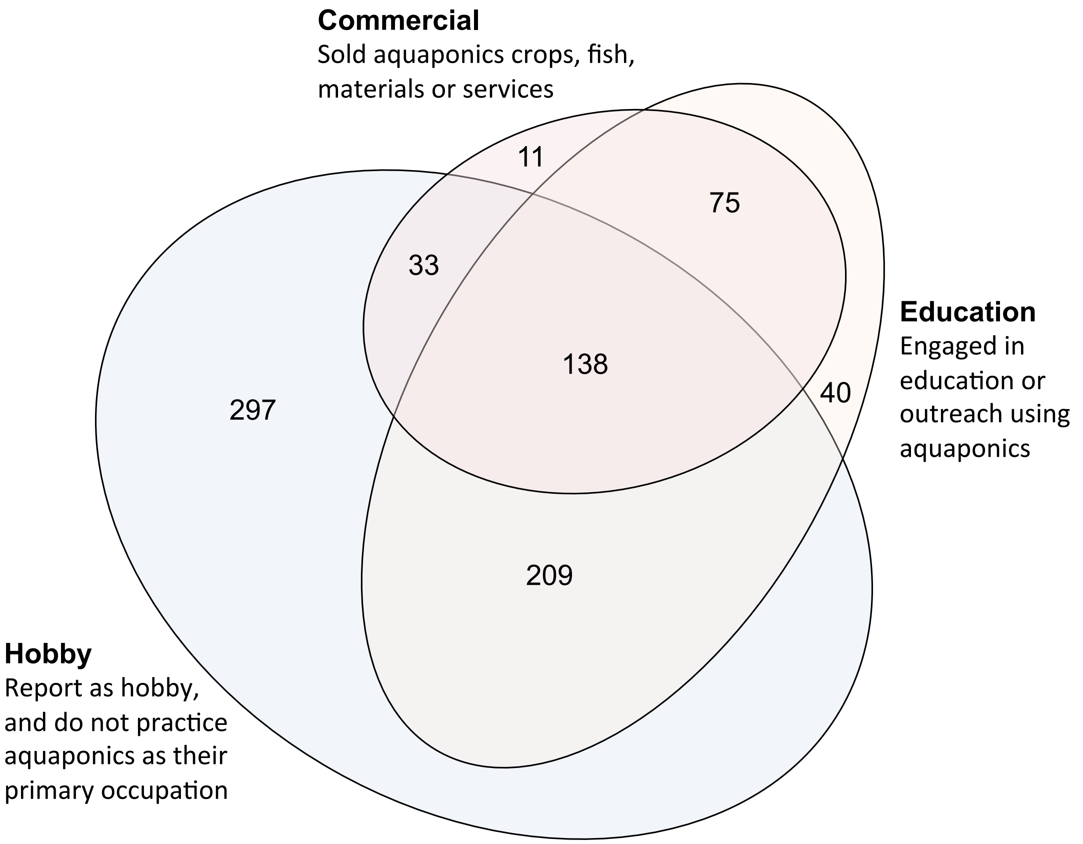

Figure S2. Venn diagram of respondents' backgrounds and experiences in aquaponics in the previous 12 months. The survey was open from June to October 2013. The Venn diagram was constructing using software eulerAPE v.3 [1], and population sample size is reported inside the ovals.

References:

1. Rodgers P, Micallef L (2013) eulerAPE v.3.
